# An Active Sensing Paradigm for Studying Human Auditory Perception

**DOI:** 10.3389/fnint.2022.892951

**Published:** 2022-05-18

**Authors:** Dardo N. Ferreiro, Valentin R. Winhart, Benedikt Grothe, Bahador Bahrami, Michael Pecka

**Affiliations:** ^1^Division of Neurobiology, Faculty of Biology, Ludwig-Maximilians-Universität München, Munich, Germany; ^2^Department of General Psychology and Education, Ludwig-Maximilians-Universität München, Munich, Germany

**Keywords:** audition, navigation, active sensing, ethology, audiomotor integration, SITh, musicality

## Abstract

Our perception is based on active sensing, i.e., the relationship between self-motion and resulting changes to sensory inputs. Yet, traditional experimental paradigms are characterized by delayed reactions to a predetermined stimulus sequence. To increase the engagement of subjects and potentially provide richer behavioral responses, we developed Sensory Island Task for humans (SITh), a freely-moving search paradigm to study auditory perception. In SITh, subjects navigate an arena in search of an auditory target, relying solely on changes in the presented stimulus frequency, which is controlled by closed-loop position tracking. A “target frequency” was played when subjects entered a circular sub-area of the arena, the “island”, while different frequencies were presented outside the island. Island locations were randomized across trials, making stimulus frequency the only informative cue for task completion. Two versions of SITh were studied: binary discrimination, and gradual change of the stimulus frequency. The latter version allowed determining frequency discrimination thresholds based on the subjects’ report of the perceived island location (i.e., target frequency). Surprisingly, subjects exhibited similar thresholds as reported in traditional “stationary” forced-choice experiments after performing only 30 trials, highlighting the intuitive nature of SITh. Notably, subjects spontaneously employed a small variety of stereotypical search patterns, and their usage proportions varied between task versions. Moreover, frequency discrimination performance depended on the search pattern used. Overall, we demonstrate that the use of an ecologically driven paradigm is able to reproduce established findings while simultaneously providing rich behavioral data for the description of sensory ethology.

## Introduction

Ethology strives to study behavior that has evolved in natural environments (Tinbergen, 1963; Lorenz, 1981). Nevertheless, behavioral neuroscience research has mainly focused on investigating phenomena by testing—often highly—trained subjects (animals, including humans) in rigid settings using predetermined test regimes. It is becoming increasingly clear that to better understand behavior, perception, and their neural underpinnings, modern neuroethological research needs to introduce more natural behavior in lab experiments (Krakauer et al., 2017; Datta et al., 2019; Gomez-Marin and Ghazanfar, 2019). Such calls to action are becoming more present in human behavioral research, underscoring how important it is to increase ecological validity (Box-Steffensmeier et al., 2022).

Accordingly, we had developed the Sensory Island Task (SIT; Ferreiro et al., 2020; Amaro et al., 2021) for animals to be tested in a lab environment with the general goal of studying sensory perception while including crucial aspects of natural behavior which would improve the ecological validity of the results. The premise of the SIT paradigm was to reintroduce the natural interdependence of movement and the sensory environment (i.e., active sensing) into laboratory settings. Specifically, we incorporated active sensing and self-motion by coupling changes in the animals’ location within a test arena with changes in the presented sensory stimuli.

In previous versions of SIT, we allowed rodents and primates to move unrestricted and thus freely explore an open field arena while their position was tracked for online closed-loop stimulation changes according to their position. Their goal was to find the “target island”, a circular area within the arena (~7% of arena surface), which randomly changes position across trials. Crucially, the only useful cue to solve the task was a change in the sensory stimulation (e.g., sound frequency) elicited when the animal enters the target island, which the animals report by staying within the island for a predefined time period (Ferreiro et al., 2020).

We recently showed that coupling SIT with neural recordings was instrumental in discovering new insights into behavior and neuronal coding in the auditory cortex of gerbils (Amaro et al., 2021; Stecker, 2021). Yet in human research, active sensing is still rarely made use of. An exemplary study by Whitton et al. (2014) tested the ability of mice and humans to use closed-loop audio-motor feedback to locate a hidden spot in a 2D arena. While mice performed this task by actually running around, humans simply moved a cursor on a computer screen *via* a joystick. This more naturalistic design (as compared to traditional forced-choice tasks) already provided valuable insights into the possibility to assess perceptual sensitivity by active sensing and encouraged us to extend our SIT paradigm to humans. Specifically, we investigated auditory frequency discrimination abilities in human subjects using real-time closed-loop feedback based on self-motion in the active sensing framework of SIT.

**Figure 1:**
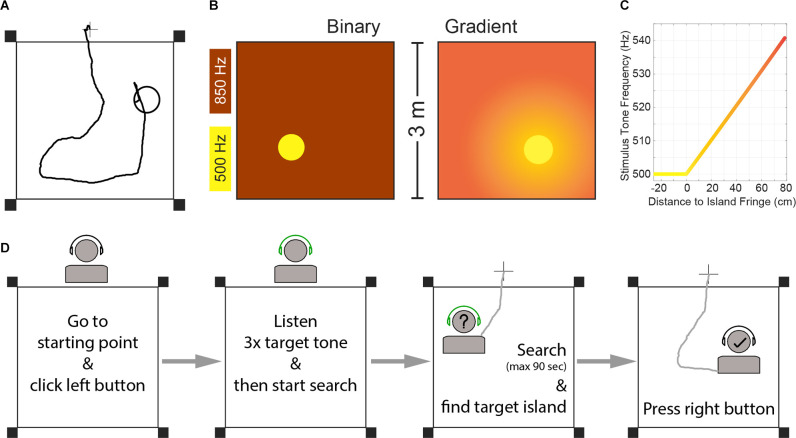
**(A)** Example trial trajectory and schematic of the experimental arena for human SIT. The circle represents the target island (island radius was 26 cm). The cross outside the arena represents the starting point of each trial. Black squares represent the position of the Oculus Rift sensors. **(B)** Schematic representation of the two task versions. **(C)** Frequency of the stimulation tone pip as a function of the subject’s distance to the island fringe for the gradient task. Negative distance values denote positions inside the island. Note: The gradient did not finish at 80 cm distance, but extended over the whole arena. See “Materials and Methods” Section for the full relationship of gradient and distance-to-island. **(D)** Schematic flowchart of trial structure.

Most characteristics of the paradigm for humans are the same as described above (i.e., free movement within an arena, stimulation coupled to subject position, no forced choice, etc.). The main differences include a bigger arena (3 × 3 meters) and position tracking *via* handheld wireless controllers, which also serve to start and finish trials (Figure 1A).

Inspired by our previous animal work, we analyzed two task versions, both based on low-frequency sound frequency perception (Figure 1B). A “binary” version, where the island and the outside area elicited two distinct frequency stimulations that are easily discriminated; and a gradient version, where the stimulation frequency depended on the distance to the target-island, inspired by the Hidden Object game that children play:

*Mary hides a toy somewhere in the room while John waits outside. John comes in and starts looking for the object while Mary indicates how close he is to the hiding place by announcing “hot” or “cold”*.

We present the implementation of the SIT paradigm in humans (SITh) as a proof-of-concept effort to reinforce the intuition that incorporating more naturalistic environments into experimental procedures in the lab is a worthwhile effort. Nonetheless, we show that with this approach it is possible to reproduce established results from classic literature without requiring extensive training of subjects. Moreover, our approach also allows for nuanced descriptions of human active sensing and the associated locomotive behavior.

## Materials and Methods

All subjects (18 total, nine males, nine females, mean age = 24.2 ± 2.4 years) who took part in the experiment showed normal audiograms between 125 Hz and 2,000 Hz.

The experimental paradigm used here for humans was inspired by previous research in our lab in animal experiments. For details about non-human versions of the paradigm, as well as for source code to run those experiments, please refer to Ferreiro et al. (2020). All relevant details for the human version are explained below.

### Procedure

Subjects moved freely within a square arena (3 × 3 meters) in search of a target island. The circular island (radius = 26 cm) covered 2.36% of the arena’s surface and its location was randomized across trials, therefore rendering the auditory stimulation as the only useful cue to find the island. While navigating the arena, subjects carried remote controllers (Oculus Rift, Meta Inc.) in their hands and wore wireless headphones through which sound stimuli were presented. To start a trial they were asked to stand over a marked point on the floor 25 cm outside of the arena (Figure 1A), and press a button on the left-hand controller. Upon trial initiation, they heard three repetitions of a 50 ms pulse of the target tone frequency. Their tracked live position (*via* the controllers) was used to determine the auditory stimulation delivered through the headphones. Participants were instructed to search for the place in the arena that would elicit the target frequency heard at the beginning of the trial, by walking naturally without unnecessary arm movements, and to press a right-hand controller button to report having found the island (See “Task Versions” below). Trials finished after participants pressed a button in the right hand controller (thus reporting having found the island) or a time limit of 90 s had been reached. Participants performed first a “binary” version of SITh and a “gradient” version subsequently, with a 5 min pause in between. Before each task version, they were allowed to practice for up to five test trials to get familiarized with the trial structure, after which they performed 30 recorded trials.

### Task Versions

Subjects performed first a binary task, in which the stimulus tone frequency was 500 Hz within the island, and 850 Hz in the rest of the arena (Figure 1B). After completing 30 trials in that task, they took a 5 min break before continuing with the gradient version. In the gradient task, the target frequency within the island was also 500 Hz, but the frequency outside of the island was proportional to the distance to the island fringe (Figure 1B), as follows:


(1) $$Frequency_{Gradient}=Frequency_{T\arg et}*1.001^{d}$$

Where *Frequency_Gradient_* is the frequency in Hz of the stimulus when the subject is outside the target island, *Frequency_Target_* is the frequency in Hz of the target island, and *d* is the distance to the island fringe.

### Experimental Setup

The arena was defined as a square of 3-by-3 m, marked on the floor of a classroom with red tape. A black cross indicated the trial starting point (Figure 1A).

The experiment was run and controlled using specifically developed code in Python. Auditory stimuli were delivered *via* Bluetooth over-ear headphones (Sennheiser HD450 BT), and consisted of 50 ms pulses of pure tones played at a repetition rate of 4 Hz. The carrier frequency of the stimulus was defined online during the experiment, as it depended on the subject’s position within the arena (See “Task Versions”).

Stimuli were cosine ramped at the on- and offset (10 ms window), and their amplitude was 60 dB SPL roved ±5 dB. The position of the subjects was determined online by averaging the tracked position of the hand-held controllers in the horizontal plane (i.e., the average position between the two hand-held controllers, which in natural walking conditions lies within the body). The controllers were part of an Oculus Rift VR set, and their position was tracked using four sensors located at the corners of the square arena. Position data were acquired from the Oculus system at 20 Hz sampling rate.

### Code Availability

The code used to run and control the experiment, which also managed the data acquisition is freely available at https://gin.g-node.org/dnferreiro/SITh.

### Data Analysis

All data were analyzed using Matlab and Python using custom scripts. For the “angle to target” (A2T) calculations, each consecutive pair of position data points in each trial trajectory was used to determine the instantaneous heading vector. Then, the A2T was determined relative to the vector containing the first of the two data points of the heading vector and the center of the target island. Therefore, an A2T of 0 degrees describes a perfectly precise heading (i.e., a step towards the center of the island). Importantly, head movement was not recorded (participants did not wear the Oculus headset). Therefore no head position dependent stimulation nor analysis were performed.

To calculate musical experience we determined the number of years each subject (based on a post-experimental questionnaire) had spent playing an instrument and/or singing. We did not distinguish by instruments, nor by formal vs. informal training. Values were normalized to the most experienced subject (Supplementary Figure 1).

### Strategy Classification

A naive human observer was asked to classify trials based on the walked trajectory patterns. Based on our extensive visual analysis of the search patterns, we identified four distinct base strategies. The observer was then instructed to assign each of the trial trajectories into five different categories, either one of the four strategies, or none. Importantly, the observer had no knowledge of the task or the study in general, he was provided only with the walked paths (similar to Figure 1A, but without the island position) and the trial presentation for classification was chronologically randomized. The observer was also allowed to flag trials for which unique categorization was perceived to be difficult. Overall, 87% of all trials were classified into one of the four strategies (73% unflagged and 14% flagged) and 13% were classified as no-strategy. The exclusion of flagged trials did not substantially alter the main findings and conclusions (data not shown).

### Statistics

Kruskal–Wallis tests were used to test for differences across search strategy distributions. Comparisons of distributions are derived from Mann–Whitney *U* tests with an alpha level = 0.05. These tests were chosen because they are non-parametric and therefore more appropriate for data distributions that deviate from normality (as can be seen from the boxplots). Comparisons of proportions are derived from Chi-square tests with an alpha level = 0.05. When applicable, multiple comparison *p*-value corrections were performed with the Holm-Bonferroni method (Holm, 1979; Aickin and Gensler, 1996).

Data distribution quantification graphs presented as boxplots use the following: black lines depict the median, filled boxes depict the first and the third quartile, error bars (whiskers) depict ± 2.7 standard deviations.

## Results

For this study, 18 normal hearing adults participated in SITh. An example trial trajectory and corresponding target island together with schematics of the arena are shown in Figure 1A. Once they started a trial, 50 ms pure tone pips were played at a repetition rate of 4 Hz. The frequency of each pip depended on the subject’s position within the arena (Figure 1B) and the version of the SITh task. Here we report on two task versions:

- “Binary”: Stimulus frequency outside the island was always 850 Hz.
- “Gradient”: Stimulus frequency outside the island was proportional to the distance to the island (Figure 1C and equation 1).

Subjects were asked to start and terminate trials autonomously, with the objective of reporting to hear the target frequency of 500 Hz. A flowchart of the basic trial structure is shown in Figure 1D (see “Materials and Methods” Section for details). Importantly, the island position was randomized across trials (see Supplementary Figure 2A for island positions across trials), and no instructions were given to the subjects regarding the task structure, island shape and size, nor potential search strategies.

**Figure 2:**
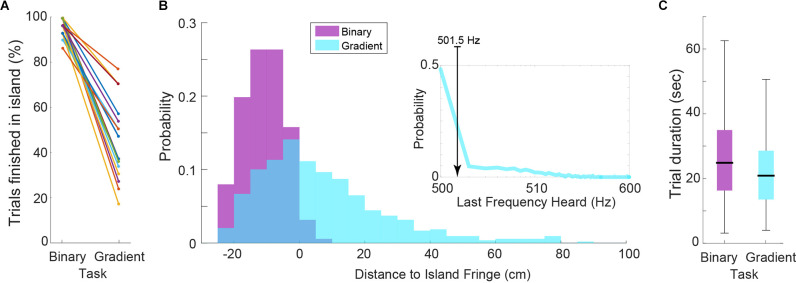
**(A)** Percentage of trials finished within the target island. Each line represents a subject. Note that while this measure is equal to performance in the binary task, it is not for the gradient task. **(B)** Histograms of the distance to island fringe for all trials across subjects for both task versions. Inset: Histogram of last frequency heard when subjects finished the gradient trials. Median of 501.5 Hz is shown. **(C)** Distributions of trial durations by task (*P* = 0.0253).

The performance of subjects, measured as a proportion of trials finished within the island, in the binary task was excellent (mean ± SD: 96.13 ± 3.80%, Figure 2A). This is not surprising given the large frequency difference between the target and non-target stimuli. By the same metric, the performance in the gradient task decreased for all subjects (44.19 ± 16.75%, Figure 2A). However, measuring performance in such a way for the gradient task does not do justice to the actual behavior and sensory perception of the subjects. Because of the stimulus frequency gradient, a hypothetical trial finishing only 10 cm away from the island means that the last frequency heard (LFH) by the subject is 505 Hz (Figure 1C). This represents a 1% difference with the target frequency which lies within the range of what traditional experiments have reported (Micheyl et al., 2012).

To better analyze the performance of subjects we computed the histograms of the radial distances to the island’s fringe (i.e., perimeter) for the final position of all trials (Figure 2B). For binary trials, this confirms the previous performance metric and further shows that the few “incorrect” trials landed very close to the island and could be related to “overshooting” (i.e., pressing the button while still moving). For gradient trials, the distance-to-island histogram is skewed towards within-island final positions. Given that within the island the stimulus frequency was always 500 Hz, we also computed the LFH histogram for the gradient trials (inset, Figure 2B). The median value of the LFH distribution was 501.5 Hz, corresponding to a 0.3% difference with the target frequency. Interestingly, this precisely matches the frequency discrimination threshold (frequency difference limen) reported by traditional “stationary” forced-choice experiments (Moore, 1973; Micheyl et al., 2012). The individual subject distributions show a median LFH <1% for 15 out of 18 subjects (Supplementary Figure 1B), proving that this metric is robust across subjects. Comparing search time between tasks showed that gradient trials tended to be shorter (Figure 2C; Mann-Whitney *U*-test double tail, *P* = 0.0253), which suggests that in fact the gradient cue helped the subjects to reach the island (or at least the general area of the island) faster.

**Figure 3:**
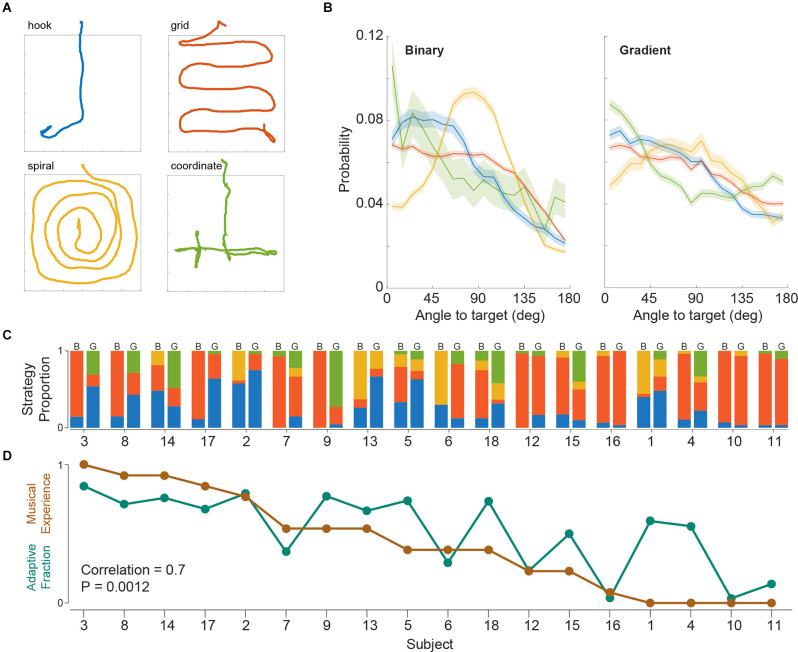
**(A)** Representative trials of the four stereotypical search strategies. **(B)** Angle to target histograms across strategies (median and 95% confidence interval). **(C)** Change in strategy use as a proportion of trials across subjects. Left and right columns per subject represent binary and gradient task versions respectively. Subject order is the same as panel **(D)**. **(D)** Fraction of trials of hook + coordinate strategies (Adaptive Fraction), and normalized years of musical experience across subjects, for the gradient task.

Given the freely moving and non-forced choice nature of SITh, we also analyzed the locomotion patterns of the subjects while searching for the target frequency. While observing task performances, we already had realized that in contrast to rodents in an identical task (Ferreiro et al., 2020), the human subjects were typically not randomly walking through the arena. After a thorough visual inspection of the recorded trial trajectories, we identified four stereotypical navigation patterns (Figure 3A). To obtain an objective description of all trials, an independent, naive observer was recruited to classify trials into the four aforementioned search strategies. Importantly, the observer was unaware of the procedure and the objectives of the study and was only provided with images of the trial trajectories without the island position (as depicted in Figure 3A). Of the entire 1,080 trials (binary and gradient), 87% were classified into one of the four strategies, while 13% remained unclassified (classified per task version: 91% for binary, 83% for gradient).

We corroborated these trial strategy classifications by an analysis of the heading angle of the walked paths (see “Materials and Methods” Section). Histograms of the angle to target revealed different profiles for each of the four strategies, thus validating the classification into these four different behavioral strategies for island search (Figure 3B). Islands were distributed uniformly within the arena across strategies (Supplementary Figure 2B), which suggests no effect of island position on strategy choice. Furthermore, we observed differences between strategies already in the initial 2 s of a trial, i.e., before the occurrence of auditory feedback (Supplementary Figure 3), suggesting that strategy selection was predetermined.

Next, we asked how strategy usage was represented across subjects (Figure 3C). We found that although a few subjects (e.g., subjects 10 and 16) had a dominant strategy and consistently used it regardless of the task version (binary or gradient), most subjects used a mixture of strategies in either task, but with altering proportions between tasks (e.g., subjects 3, 4, 9, 14, and 18). To analyze whether there was a consistent population level change across subjects involving any particular strategy, we performed Mann-Whitney tests comparing the strategy proportions across subjects in binary and gradient trials (*P*_hook_ = 0.11, *P*_grid_ = 0.09, *P*_spiral_ = 0.38, *P*_coordinate_ = 0.00011). This revealed that the coordinate strategy was significantly more adopted when switching to the gradient task.

Given that the hook and coordinate strategies both make use of the online feedback provided by the gradient stimulation (as opposed to the other more rigid, systematic strategies), we wondered why some subjects opted to switch to these adaptive strategies more often than others. Since the changes in frequency near the island fringe are rather small (relative to perceptual levels) during the gradient task, it would be beneficial if subjects were able to reliably identify the target frequency. Such identification could have been favored by a high familiarity with musical training and/or exposure. Therefore, we tested whether the usage of these “adaptive” strategies in the gradient task was associated with the subjects’ musical experience (Figure 3D and see “Materials and Methods” Section). We did in fact find a correlation between both measures, which suggests that the greater exposure to music subjects had, the more they would be inclined to use an adaptive strategy (Pearson correlation = 0.7; *P* = 0.0012).

**Figure 4:**
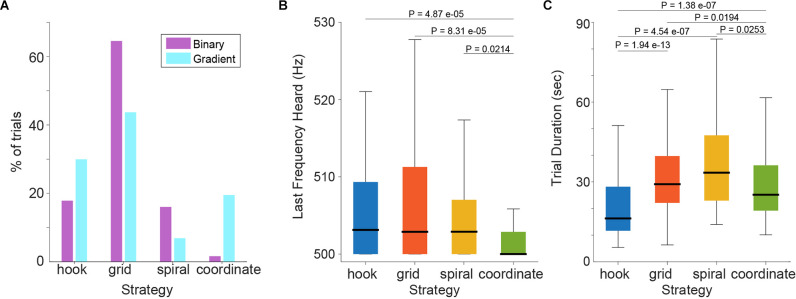
**(A)** Change in strategy use as a percentage of total trials in the two task versions. **(B)** Last frequency heard by subjects and; **(C)** Trial duration across strategies, in the gradient task.

Distributions in strategy use on the population level are summarized in Figure 4A. After switching to the gradient condition, grid and spiral strategies were used less, and coordinate and hook strategies were more frequent. This change is expected because grid and spiral strategies entail systematic surface coverage, while coordinate and spiral incorporate the sensory feedback during the active search.

Based on these findings, we wondered whether the different search strategies were correlated with different levels of frequency discrimination performance. We, therefore, computed the LFH across strategies (Figure 4B, compare Figure 2B). Statistical analysis showed that the four strategies differed in their LFH distributions (Kruskal-Wallis test, *P* = 2.1463e-04; Median values: hook = 503.1 Hz, grid: 502.9 Hz, spiral = 502.9 Hz, coordinate = 500 Hz). This analysis thus demonstrates that the locomotive behavior of subjects was correlated with the auditory frequency discrimination performance and suggests that the best performance was achieved when using the coordinate strategy. Performing statistical comparisons of the coordinate vs. each of the other three strategies (Mann-Whitney *U*-tests single tail, Holm-Bonferroni corrected; *P*_coordinate-hook_ = 4.8714e-05, *P*_coordinate-grid_ = 8.3156e-05, *P*_coordinate-spiral_ = 0.0214) confirmed that the coordinate strategy, which was spontaneously adopted by subjects when confronted with the gradient task, is associated with significantly better frequency discrimination performance. We further looked into whether practice influenced the choice of strategy by subjects, but did not find a pattern. Interestingly, while the use of adaptive strategies in the gradient task correlated with the musical experience across subjects (Figure 3D), the frequency discrimination performance on average did not (Supplementary Figures 1A,B). We did find a mild correlation between LFH and musical experience when including all the trials instead of comparing only with the median LFH (Supplementary Figure 1C). However, when removing subjects with zero musical experience (four subjects total), this correlation was lost (Supplementary Figure 1D).

We also compared trial duration times across strategies (Figure 4C; Kruskal-Wallis test, *P* = 5.0732e-15; Median values: hook = 16.3 s, grid = 29.1 s, spiral = 33.5 s, coordinate = 25.2 s) and observed that the fastest strategy was the hook, followed by the coordinate (Mann-Whitney *U*-tests single tail, Holm-Bonferroni corrected; *P_hook-grid_ = 1.9402e-13*, *P*_hook-spiral_ = 4.5475e-07, *P*_hook-coordinate_ = 1.3890e-07, *P*_coordinate-spiral_ = 0.0253, *P*_coordinate-grid_ = 0.0194).

The data thus indicate that on average trials with adaptive strategies finished faster compared to non-adaptive strategy trials. Interestingly, between the two adaptive strategies, hook trials were even faster than coordinate trials, yet also showed worse frequency performance. These observations together suggest that coordinate strategy users may have invested time “subjectively verifying” the detection of the target frequency. In line with this assumption, we noticed that subjects regularly performed small probing movements (rocking or taking a step back and forth) near or inside the island to determine their perceptual discrimination threshold (i.e., they were not able to detect any further change in frequency) before pressing the button to indicate the end of the trial. Quantification of this fine-tuning behavior (respective trials were flagged by the same naive observer as for strategy classification) confirmed that it was indeed more frequent for coordinate trials during the gradient task (Fraction of trials showing fine-tuning: hook = 0.32, grid: 0.33, spiral = 0.16, coordinate = 0.5000; Chi-square test, Holm-Bonferroni corrected; *P*_coordinate-hook_ = 0.02646, *P*_coordinate-grid_ = 0.02465, *P*_coordinate-spiral_ = 0.00492, *P*_hook-spiral_ = 0.16327, *P*_hook-grid_ = 0.75306). Thus, it appears that strategy use and resulting discrimination thresholds were also related to the likelihood to optimize trial performance. Interestingly, we also found that subjects that exhibited more trials with fine-tuning behavior also showed a tendency for lower median LFH in the gradient task (Supplementary Figure 4A). This tendency was present even when excluding coordinate trials (Supplementary Figure 4B). In contrast, no correlation was found between fine-tuning behavior and the standard deviation of the LFH (as a measure of performance variability) nor with musical experience (Supplementary Figure 4).

Together, the data show that performance levels in frequency discrimination obtained by using closed-loop feedback during voluntary self-motion in subjects with only a few minutes of training can match those reported using traditional forced-choice paradigms and highly trained participants (Micheyl et al., 2012). Moreover, subjects spontaneously develop various distinct locomotion strategies for task completion, which differ in their average performance outcome.

## Discussion

Inspired by contemporary calls to action to bring together laboratory experiments and natural ethological investigations, we investigated the potential of experimental paradigms based on closed-loop audio-motor feedback for application in human psychophysical research.

We tested a new procedure to study sensory perception in humans, which would resemble and encourage natural exploration behavior. Traditional laboratory settings usually predetermine the strategy that can be used to discriminate between stimuli and require *post-hoc* decision-making (i.e., forced-choice after having listened to all alternatives). In contrast, SITh is based on unrestricted self-motion to modulate the test stimuli and thereby allows subjective optimization of performance. Specifically, we incorporated active sensing during self-motion by coupling changes in the subjects’ location within a test arena with changes in the presented sensory stimuli and allowed subjects to manipulate the stimulus until they were satisfied with their perceived performance. We focused on sound frequency discrimination, which is a well-established measure of auditory perception. To this end, we implemented a frequency gradient stimulation within an arena, which served as immediate feedback for their navigation.

Due to the attentional burden on cognitive processes imposed by the more naturalistic environment (i.e., locomotion, visual input, feedback evaluation, guided action, etc.), we expected worse discrimination thresholds for our naive subjects compared to those reported from classic experiments with expert listeners. Surprisingly, we obtained a frequency discrimination threshold of 0.3% on average across all subjects, which matches the results from “stationary” subjects comparing tone pairs in alternative forced-choice tasks (Micheyl et al., 2012). This is surprising given that we allowed subjects only a few minutes to familiarize themselves with the task, while typically highly experienced subjects are used and longer data acquisition periods are needed. This suggests that performing unrestricted experiments might be advantageous for accessing marginal perceptual limits by allowing a range of natural behaviors that may assist the performance under investigation. Real-time sensory feedback and cross-sensory cueing have been shown to be favorable in human psychophysical experiments (Whitton et al., 2017; Clayton et al., 2021). Notably, Whitton et al. (2014) tested subjects on signal-to-noise discrimination by providing closed-loop audio-motor feedback *via* joystick control to move avatars on a screen and reported a similar fine-tuning behavior of subjects when being close to the perceptual threshold, suggesting that this behavior is naturally occurring during active sensing. Participants in our study performed actual translational movements inside an arena. This “real-word” movement may have additional advantages since self-motion and its resulting modulations may aid the interpretation of sensory cues in natural settings (Freeman et al., 2017; Willett et al., 2019).

By analyzing the locomotion behavior of the subjects, we revealed that they spontaneously adopted stereotypical navigation patterns, which we categorized into four search strategies. Interestingly, the analyzed strategies seem to differ in their use of the gradient information. The “spiral” and the “grid” strategies are characterized by systematic surface coverage at the expense of not following gradient cues, while “hook” and “coordinate” describe stimuli adapting behavior, as known to happen for head movement in sound localization (Pollack and Rose, 1967). Consistent with the ability of the strategies to incorporate the gradient cues into the navigation, subjects were more likely to use the coordinate and hook strategies after switching to the gradient condition. These two strategies were characterized before as “gradient descent” and “coordinate descent” in a signal-to-noise ratio gradient task performed by stationary humans *via* a joystick (Whitton et al., 2014). Here we find that the coordinate strategy enabled a smaller frequency discrimination threshold (Figure 4B), presumably because it better exploits the gradient cue, while the hook strategy is characterized by rapid (and likely less diligent) honing into the gradient descending frequencies. Accordingly, the hook strategy exhibited the shortest trial durations on average, but larger discrimination thresholds compared to the coordinate strategy. Similarly, the coordinate strategy seems to also save time compared to the systematic (non-adaptive) searches, but without sacrificing accuracy like the hook strategy. These findings can be partially explained by the differences in “fine-tuning behavior”, i.e., the fraction of trials in which subjects performed small corrective movements to more exactly determine the location of the target island/last frequency heard before finishing the trial. Such behavior was significantly more frequent in coordinate trials. Accordingly, frequency discrimination thresholds were significantly smaller when using this strategy (when averaging across all subjects and trials). Moreover, a higher proportion of fine-tuning behavior tended to correlate with better discrimination performance across subjects, even when excluding the coordinate trials (Supplementary Figure 4). This suggests that performance during active sensing is affected both by the global search strategy and by local adjustments. In the future, it will be interesting to determine whether these advantages of strategy use also pertain to the single-subject level, i.e., do perceptual thresholds of individual subjects change significantly when restricting the search to specific strategies? Conversely, could restricting subjects to the use of a specific strategy minimize performance variability and provide further insight into the capabilities of human perception during active sensing?

Interestingly, while formal musical training has been shown to improve frequency discrimination performance (Micheyl et al., 2006), we did not find a significant gradual relationship between performance and informal musical experience (Supplementary Figures 1A,B). Nonetheless, our data suggests that having no musical experience entails a high likelihood of performing worse than subjects with experience (Supplementary Figures 1C,D). Musicality of subjects also did correlate with their choice of the search strategy. More musically experienced subjects tended to use “adaptive” strategies (i.e., “coordinate” and “hook”; Figure 3D). This suggests that musical experience may influence the subjects’ disposition to interact with the available gradient cues, perhaps resembling the natural audiomotor feedback present while singing or playing an instrument. Given that we did not distinguish between formal and informal musical experience, it remains interesting for future studies to determine whether they exert different influences both in the active sensing behavior as well as in the frequency discrimination performance.

In summary, our results provide proof-of-concept for the suitability of SITh for conducting sensory perception studies during naturalistic active sensing behavior. Future studies on the influence of stimulation parameters (such as frequency and tone duration) on auditory perception during active sensing are readily obtainable with the current setup configuration. Furthermore, SITh allows physiological investigations of the neural mechanisms underlying auditory feedback-guided navigation by incorporating mobile EEG recordings (for an example on visual perception see Dowsett et al., 2020). Valuable insights could also be gained by comparing behavioral and physiological results obtained in real-world locomotion with cursor-based and/or virtual reality movement. Since the code to run SITh is freely available and several parameters can be readily adapted (e.g., size of the islands, stimulus parameters, free field speakers instead of headphones, etc.), the experimental paradigm provides superb versatility and should foster further research on the interplay between hearing, self-motion and its neural correlates in the future.

## Data Availability Statement

The raw data supporting the conclusions of this article will be made available by the authors, without undue reservation.

## Ethics Statement

The studies involving human participants were reviewed and approved by the ethical board of the Ludwig-Maximilians-Universität medical center and carried out in accordance with the ethical principles of the world medical association for research involving human subjects (Declaration of Helsinki). The patients/participants provided their written informed consent to participate in this study.

## Author Contributions

DNF and MP conceived the study, designed and conducted the experiments. DNF and VRW analyzed the results. DNF designed and generated the figures. DNF wrote the first draft of the manuscript, and MP, BB, and BG made contributions. All authors contributed to the article and approved the submitted version.

## Conflict of Interest

The authors declare that the research was conducted in the absence of any commercial or financial relationships that could be construed as a potential conflict of interest.

## Publisher’s Note

All claims expressed in this article are solely those of the authors and do not necessarily represent those of their affiliated organizations, or those of the publisher, the editors and the reviewers. Any product that may be evaluated in this article, or claim that may be made by its manufacturer, is not guaranteed or endorsed by the publisher.

## Supplementary Material

Supplementary Figure 1 **(A)** Normalized years of musical experience. **(B)** Distribution of last frequency heard across subjects. Both panels: the subjects are ordered by ascending median LFH. Pearson correlation of musical experience and median LFH: rho = -0.38; p = 0.12. **(C)** Pearson correlation of musical experience and LFH across all trials. **(D)** Same as **(C)** but removing four subjects with zero musical experience.

Supplementary Figure 2 **(A)** Location of all the islands by task. **(B)** Location of the islands in the gradient task by the strategy used in the corresponding trial.

Supplementary Figure 3 The first 2 s of the subjects’ path for every gradient trial, separated by strategy. The black line depicts the border closest to the starting point. Note that strategy-specific trajectories can be observed even before having sensory feedback (i.e., before crossing the black line).

Supplementary Figure 4 Fine-tuning behavior tends to correlate with discrimination performance when analyzing all trials **(A)** and when excluding coordinate trials **(B)**. Panels depict the correlation of fine-tuning proportion with normalized musical experience, median last frequency heard or standard deviation of the last frequency heard as a measure of the variability of the performance, respectively. Each dot represents a subject. All panels, y axis: proportion of gradient trials which showed fine tuning behavior. Pearson correlation coefficient and p-values are given in the figure.
