# Supplementary figures and images for: An Active Sensing Paradigm for Studying Human Auditory Perception
Source: Front Integr Neurosci. 2022 May 18;16:892951. doi: 10.3389/fnint.2022.892951 (PMC9159912; doi:10.3389/fnint.2022.892951)

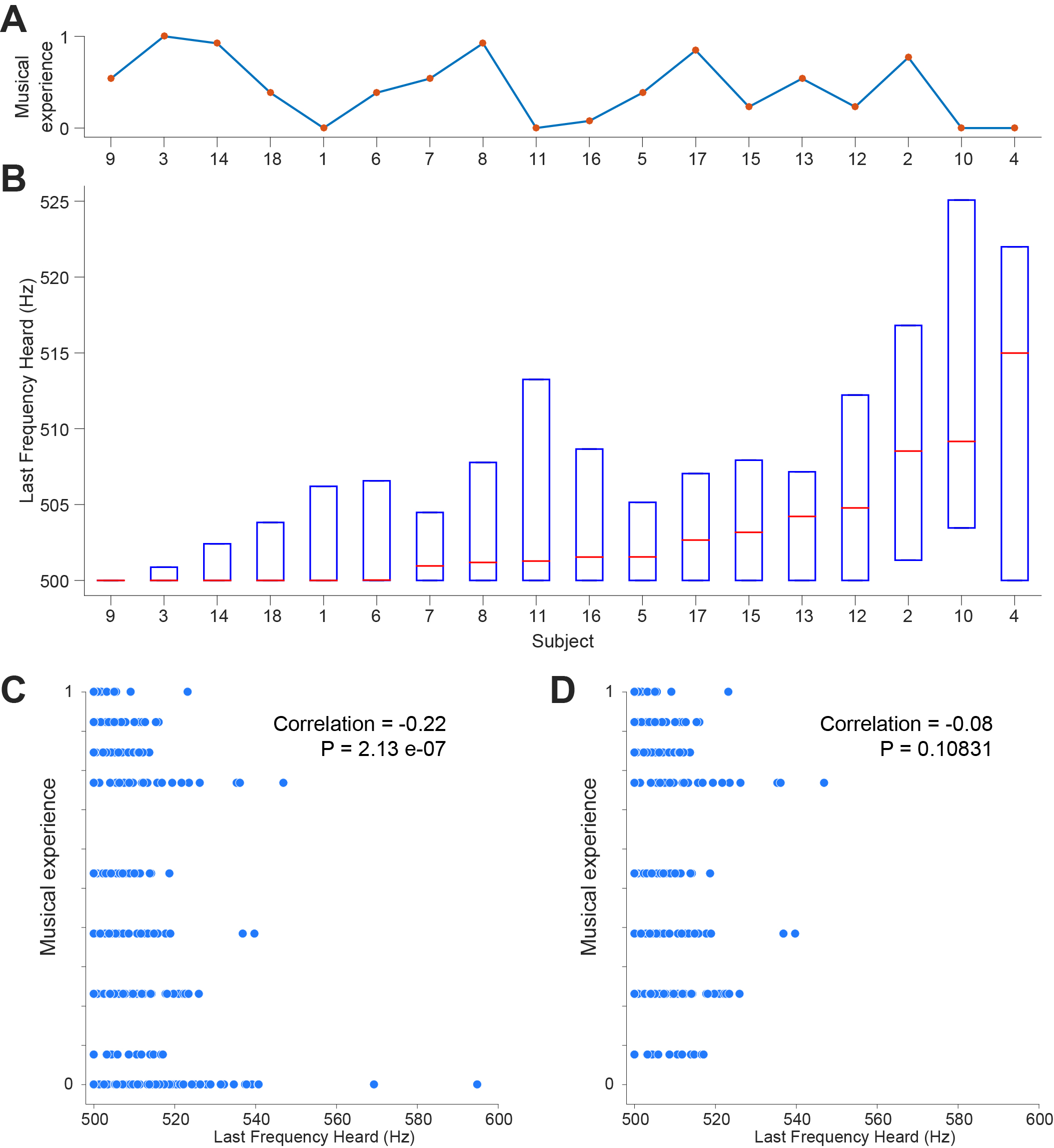

Supplement: Supplementary Figure 1 — (A) Normalized years of musical experience. (B) Distribution of last frequency heard across subjects. Both panels: the subjects are ordered by ascending median LFH. Pearson correlation of musical experience and median LFH: rho = -0.38; p = 0.12. (C) Pearson correlation of musical experience and LFH across all trials. (D) Same as (C) but removing four subjects with zero musical experience. [file Image_1.jpeg]

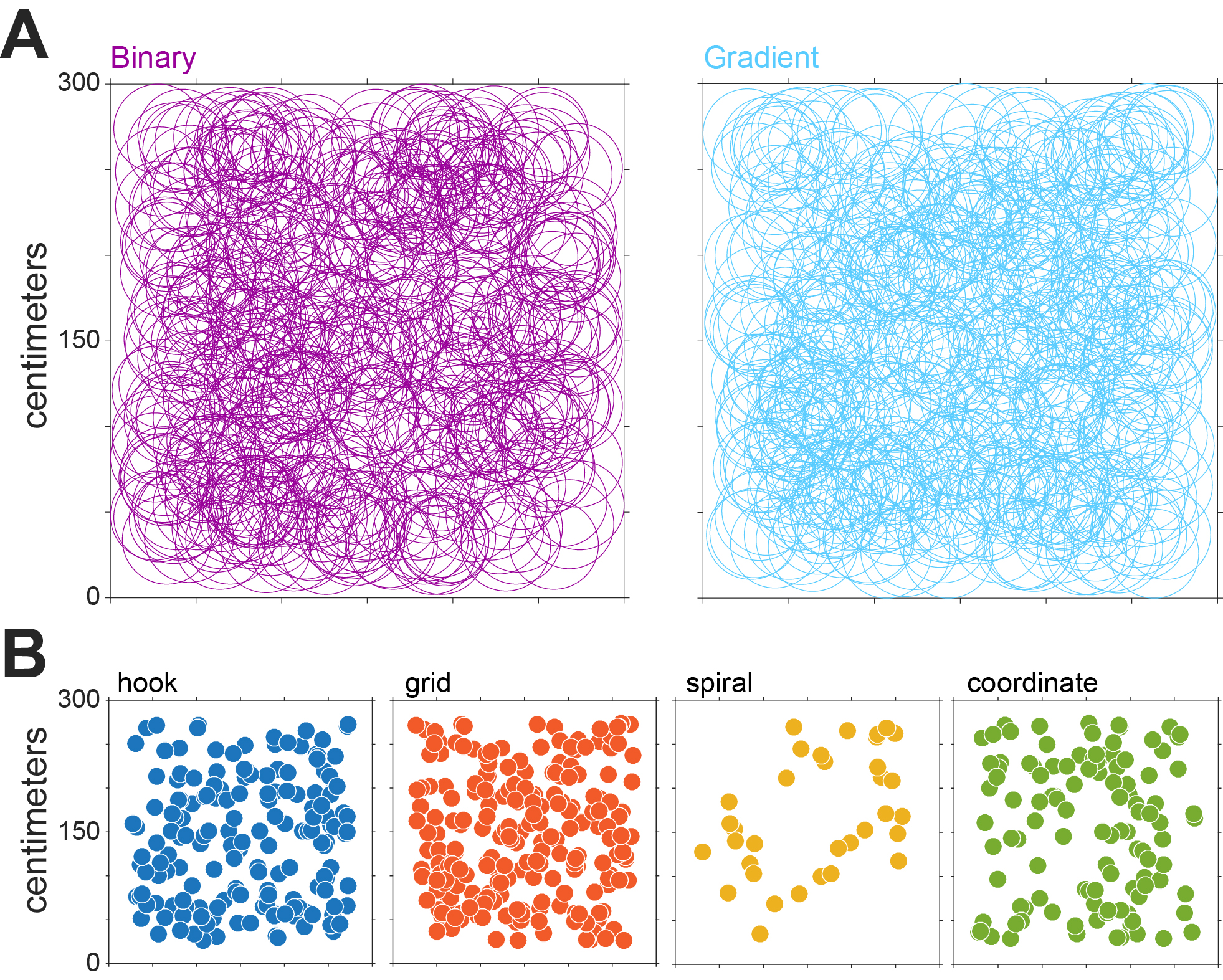

Supplement: Supplementary Figure 2 — (A) Location of all the islands by task. (B) Location of the islands in the gradient task by the strategy used in the corresponding trial. [file Image_2.jpeg]

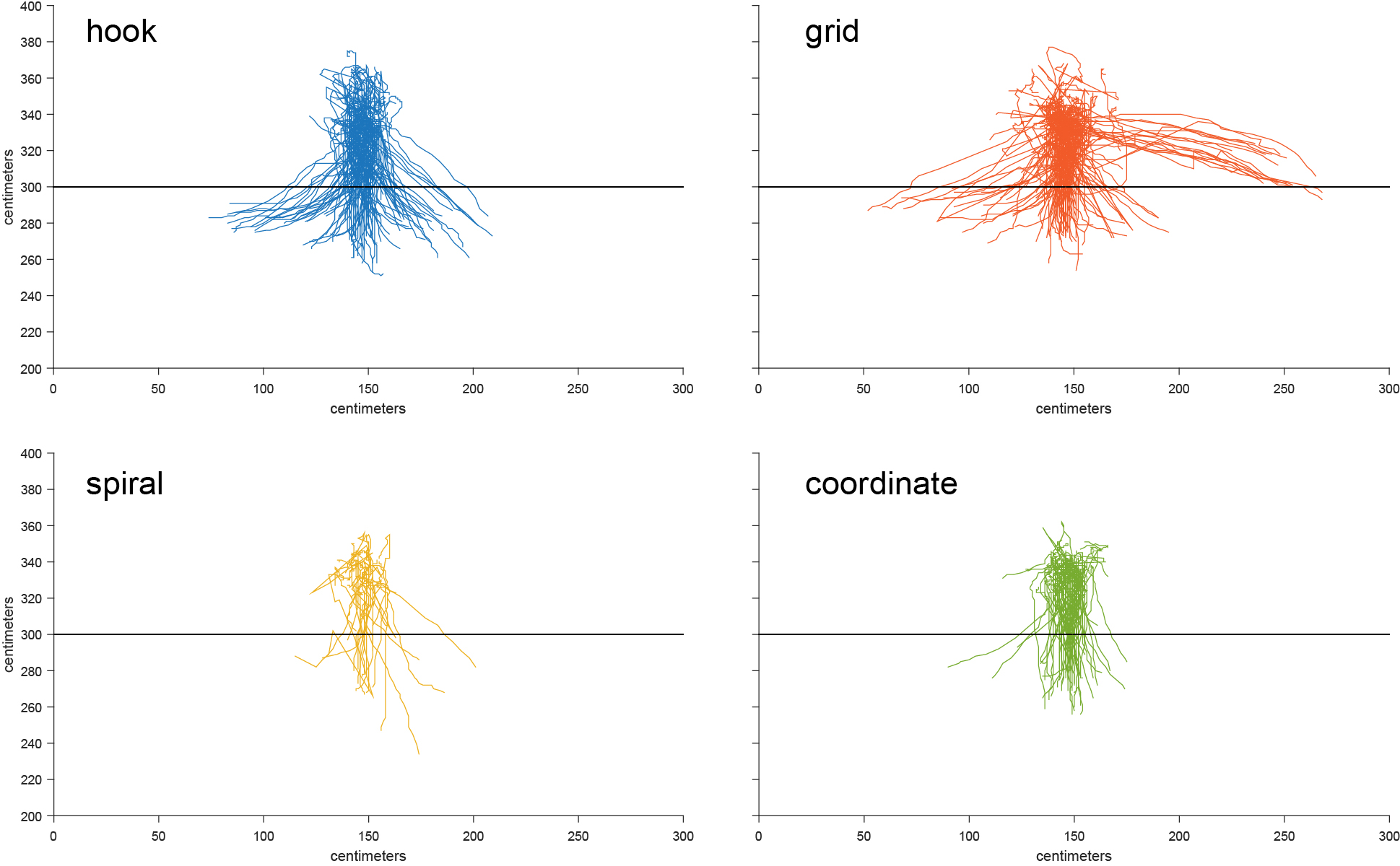

Supplement: Supplementary Figure 3 — The first 2 s of the subjects’ path for every gradient trial, separated by strategy. The black line depicts the border closest to the starting point. Note that strategy-specific trajectories can be observed even before having sensory feedback (i.e., before crossing the black line). [file Image_3.jpeg]

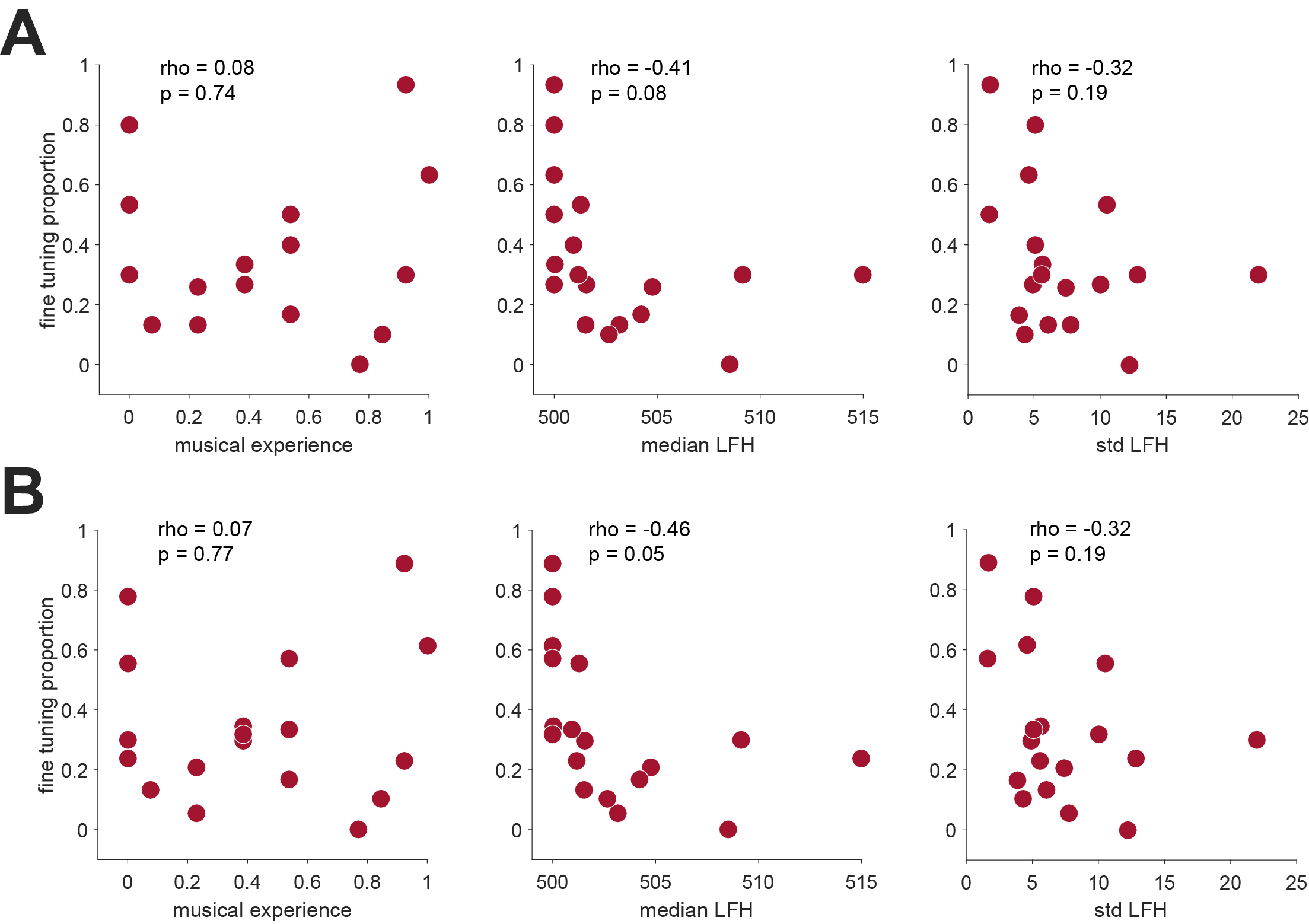

Supplement: Supplementary Figure 4 — Fine-tuning behavior tends to correlate with discrimination performance when analyzing all trials (A) and when excluding coordinate trials (B). Panels depict the correlation of fine-tuning proportion with normalized musical experience, median last frequency heard or standard deviation of the last frequency heard as a measure of the variability of the performance, respectively. Each dot represents a subject. All panels, y axis: proportion of gradient trials which showed fine tuning behavior. Pearson correlation coefficient and p-values are given in the figure. [file Image_4.jpeg]
